# The art of mabisi production: A traditional fermented milk

**DOI:** 10.1371/journal.pone.0213541

**Published:** 2019-03-14

**Authors:** Himoonga Bernard Moonga, Sijmen E. Schoustra, Anita R. Linnemann, Elias Kuntashula, John Shindano, Eddy J. Smid

**Affiliations:** 1 Laboratory of Food Microbiology, Wageningen University, Wageningen, The Netherlands; 2 Laboratory of Food Quality & Design, Wageningen University, Wageningen, The Netherlands; 3 Laboratory of Genetics, Wageningen University, Wageningen, The Netherlands; 4 Department of Food Science & Nutrition, School of Agricultural Sciences, University of Zambia, Lusaka, Zambia; 5 Department of Agricultural Economics & Extension, School of Agricultural Sciences, University of Zambia, Lusaka, Zambia; Agricultural University of Athens, GREECE

## Abstract

Fermented dairy products can be rich in beneficial microbes and one such product with potential is mabisi. Mabisi is a traditional fermented milk product from Zambia made through spontaneous fermentation of raw milk at ambient temperature using a calabash (gourd), clay pot, plastic or metal container. The fermentation takes about 48 hours after which the product is stirred and ready for consumption. This study was aimed at determining the types of production methods of mabisi and identifying the critical production process parameters. A survey was conducted using interviews and observations to determine the existing production practices/technologies and to capture indigenous knowledge on mabisi production in nine provinces of Zambia. We found seven different production methods which we coined; tonga, thick-tonga, illa, barotse, backslopping, cooked and creamy types. Interestingly, the tonga-type mabisi was produced throughout the country by different ethnic groups. The main process parameters were found to be fermentation time and temperature, type of containers, presence/absence of backslopping, agitation, heating and cooling, removal of whey and addition of raw milk. And further found that mabisi is a versatile product consumed with a wide variety of foods. This basic information is crucial for production process optimisation and microbial communities dynamics studies.

## 1. Introduction

Most fermented food products found on the market originate from traditional recipes that have evolved and have been optimised over the years. A good example is cheese of which the production can be traced back to 5,500 BC in present day Poland, where filtering clay pots were found with remnants of milk fat, [1, 2]. The production of fermented milk products has from that time evolved in different regions and countries in Europe leading to various specific types of cheeses for example Parmesan cheese from Parma in Italy, Swiss cheese from Switzerland, Gouda from The Netherlands, Brie from France and Cheddar from the UK.

Sub-Sahara Africa is not renowned for cheese production and does not have famous indigenous fermented milk products such as yoghurt or kefir. However, there are definitely traditional popular fermented milk products in most countries, which are still produced using traditional methods at household level and for which production processes have not or scarcely been documented. An example is mabisi, a traditional fermented milk product from Zambia, which according to Schoustra [3] is made by spontaneous fermentation of raw milk at room temperature for 48 hours in a calabash (gourd) then stirred/sieved before consumption.

Zambia is a vast country with 10 provinces and 73 tribes (ethnic groups). It has a total cattle population of 4,984,909 out of which 70% are under traditional (small-scale) production system while 30% are under a commercial (large-scale) production system [4]. The total estimated annual milk production is 1,179,000,000 litres [4] but less than 50% is processed by dairy companies, [5, 6]. The rest is sold through informal channels either as fresh raw milk or as mabisi which is also referred to as ‘sour milk’ and of which the total annual production is also unknown. The low commercial milk processing levels are mainly because most dairy companies are situated in urban areas (large cities). In contrast, the highest cattle and milk production is in the rural areas, where the road infrastructure is poor and access to cold storage facilities is limited due to erratic or inadequate power supply. Mabisi production is one way of preserving milk in these areas. Moreover, it is a popular product because of its taste and other organoleptic properties, and is mainly consumed as a beverage or with the staple maize meal thick porridge.

Other studies on traditional fermented milk in Africa have revealed products such as *amasi* in Zimbabwe and in the Republic of South Africa (RSA) [7], *Omashikwa* in Namibia [8, 9], *Mursik* in Kenya [10], *Kivuguto* in Rwanda[11], *Nunu* in Ghana [12] and *Aewsso* in Ethiopia [13]. Most of these studies were conducted in specific regions or parts of those countries where the products are mostly produced and consumed. These studies highlight that for most traditional products, many variations of processing exist and this processing relies on traditional knowledge, for which in some cases modern standardized procedures have been developed.

Mabisi is traditionally produced in the western, central and southern parts of Zambia, where a larger proportion of cattle raised under the traditional production system are located. However, with internal migration and promotion of dairy production by the Government and non-governmental organisations (NGOs) in other regions, the likelihood that mabisi production has spread to those regions has not been ascertained. The drivers of internal migration have largely been the search for fertile agricultural land in areas with high rainfall (towards the northern regions) as well as employment which lead to mixing of different ethnic groups and exchange of cultural practices. A previous study by Schoustra [3] reported that only one production method of mabisi making was practiced in 6 sites located in southern (3 sites), central (2 sites) and central-north (1 site) parts of the country. The study excluded one traditional mabisi production region which is the western part and also a non-traditional mabisi production region, the eastern part of the country. Overall, there is currently limited documentation of the different traditional technologies that are used for producing mabisi.

Therefore, this study aimed at determining and documenting the different mabisi production practices and technologies throughout the country and to analyse how they are influenced by region and ethnicity. The study further elucidates the key processing steps and parameters that are critical to product quality and safety, evaluates the hygiene practices as well as uses of the final product. We envisaged that this comprehensive approach of gathering indigenous knowledge on traditional fermentation practices of milk when added to other regional contributions from the different parts of Africa will give an overall picture of these products for potential improvement and up-scaling of production to the levels of other renowned fermented milk products in the world.

## 2. Material and methods

### 2.1 Study area and sampling

A cross-sectional study was conducted country-wide covering 9 out of 10 provinces of Zambia. Since mabisi is mostly produced in Southern, Western and Central provinces, more districts were sampled in those provinces as shown in Table 1. Out of the rest of the other provinces, Eastern province had more districts sampled despite not being a renowned region for producing mabisi but because of its high cattle population. Lusaka and Copperbelt province are more cosmopolitan and urban with the former being the capital city and the latter being a mining region. The selection of the districts was based on information on dairy production activities and cattle population provided by the Ministry of Fisheries and Livestock (MFL) officials as shown in Table 1. Luapula province was excluded due to low cattle population (21,564) compared to rest [4, 6].

**Table 1:** Study sites, cattle population and dairy cooperatives per province.

Province	Cattle population	No. of Dairy Cooperatives interviewed	Districts sampled
Western	890,288	2	Kaoma, Mongu, Kalabo, Senanga, Sioma, Sesheke
Southern	2,105,890	6	Choma, Sinazongwe, Namwala, Kalomo, Zimba, Monze
Eastern	844,839	1	Lundazi, Chipata, Sinda
Central	576,169	2	Chibombo, Mkushi, (Mumbwa, Itezi-tezi)*
Copperbelt	69,616	2	Luanshya, Chingola
North-western	93,508	1	Solwezi, Kabompo, Zambezi
Muchinga	175,801	2	Mpika, Isoka
Northern	79,044	1	Mbala
Lusaka	128,190	1	(Chongwe)*

Source for cattle population: Ministry of Fisheries & Livestock (MFL), 2017

*For districts in brackets, only focus group discussions were conducted.

### 2.2 Data collection

The data were collected using structured questionnaires (S1 Questionnaire) which were administered in one-on-one interviews by the researchers in English and/or local languages that the respondents were most familiar with. At least 30 questionnaires were administered per district and they had several sections which included socio-demographic information, mabisi production methods, processing parameters and hygiene practices. Districts with fewer respondents were combined with proximal districts within the same province. The respondents were selected randomly from a list of livestock farmers in each veterinary camp (VC) within the district that was selected based on recommendations given by the Ministry of Fisheries and Livestock personnel. Districts are divided into VCs for administration purposes. Focus group discussions (FGDs) were conducted with farmer groups of 8–12 people and 1–2 FGDs per district were carried out depending on the number of respondents available. Semi-structured interviews with key informants who included chairpersons of dairy cooperatives, managers of milk collection centres (MCCs) and local Headmen or Chiefs as well as Ministry of Fisheries and Livestock officers at provincial and district level were also carried out using a set of questions (S1 Checklist) on the detailed production process of mabisi, product safety and uses. Data were also collected by observation of production practices and equipment used. In all cases, consent for the interviews was formally requested and granted. In addition, ethical approval from ethical review committees both at the University of Zambia (UNZA) and Wageningen University was granted for this study.

### 2.3 Data analysis

The data from the questionnaires were entered in a datasheet in IBM SSPS statistics version 22 software. Then, the data were analysed using both IBM SSPS statistics 22 and Microsoft excel software for descriptive statistics of mean, percentage as well as correspondence analysis (CA).

## 3. Results

The main objective of this study was to determine the traditional production practices of mabisi used by local households (HH) and dairy cooperatives. Out of the 537 respondents, 76% were male and 24% were female mainly because mabisi is largely produced by the households that own cattle (93%) which mostly belong to the men. A higher proportion of these HH were headed by men (88%) and the highest HH size was between 6–10 (43%). Most respondents were married (83%) with the dominant (24%) age group being between 40–49 years as shown in Table 2.

**Table 2:** Demographic information of the respondents.

**Parameter**	**Gender**		**Marital status**				**Households with Cattle**		**Household head**	
Descriptor	Male	Female	Single	Married	Divorced	Widow(er)	Yes	No	Male	Female
%	76	24	9	83	2	6	93	7	88	12
**Parameter**	**Household size**			**Age (years)**						
Descriptor	0–6	6–10	>10	<18	18–29	30–39	40–49	50–59	>60	
%	33	43	24	1	13	21	24	20	21	

### 3.1 Mabisi production methods

This study revealed seven (7) distinct methods of mabisi processing, each yielding a distinct type which are described in this section.

#### 3.1.1 Tonga & thick tonga type

**Fig 1:**
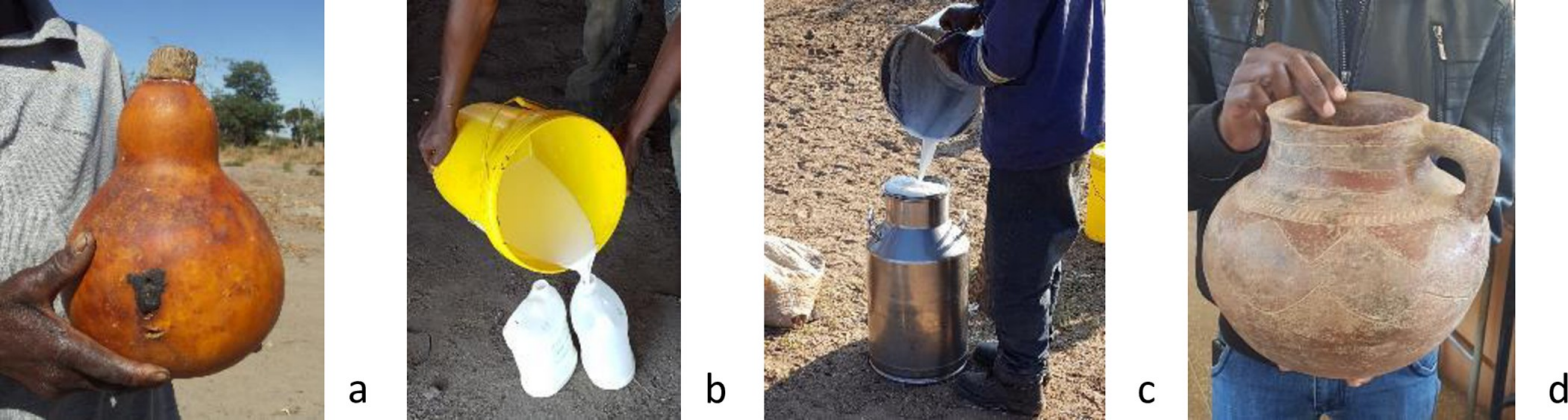
Types of fermentation containers used. These include: a. calabash, b. plastic bucket & 2.5 litre containers, c. metal container and d. earthen pot.

The production of mabisi begins with milking, the raw milk is milked by hand into a bucket which is either made of plastic or metal. Traditionally, gourds (calabash) with large openings and earthen pots were used for this purpose, [7, 14]. The raw milk is then sieved mostly using a plastic tea strainer (64%), stainless steel strainer (21%) or a cloth (7%). The plastic tea strainer is the most popular because it is cheap and readily available in most local markets. The raw milk is then poured into a plastic bucket (21%) or plastic container with a smaller opening (56%), metal can or pot (12%), calabash (gourds)(6%), or an earthen pot (nongo)(2%) covered with a lid and placed in a cool place in the house in most cases (91%) as shown in Fig 1. To speed up the fermentation, the container can be put in direct sunlight (3%) during the day or placed in the kitchen (2%) especially in the colder months of June and July when ambient temperatures (10–22°C) are lower. Sometimes, the milk is incubated outside the house in the shade but the FGDs revealed that a cold waterbath may be used during the hot months of September–November (20–30°C).

**Fig 2:**
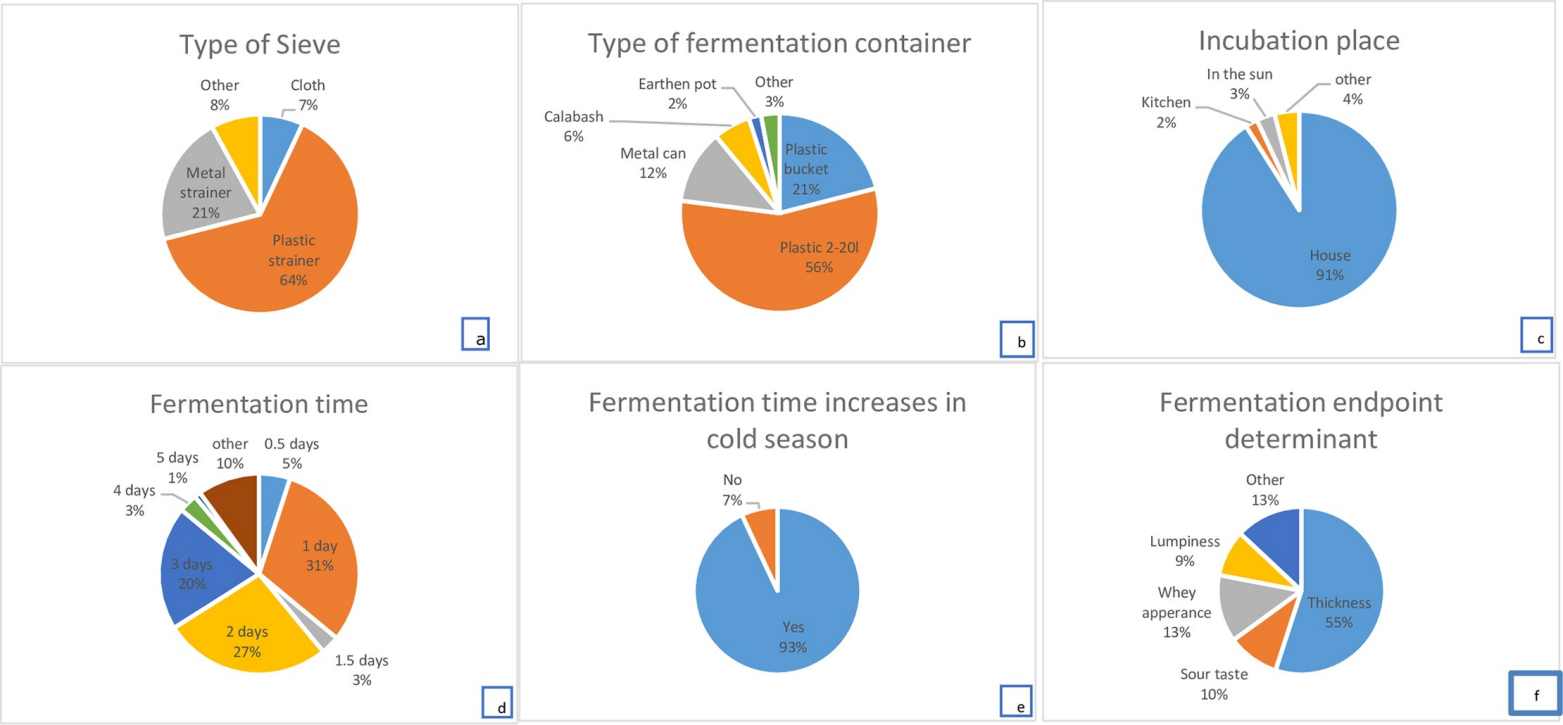
Proportions of key mabisi production process parameters of the respondents’ responses. The parameters include the following: a. type of sieve used, b. type of fermentation container, c. incubation place during fermentation, d. period of fermentation (time), e. increase in fermentation time (period) during the cold season, and f. the determinants of the end fermentation.

The container is left to ferment spontaneously for mostly 1–3 days (86%) but it can actually go up to 7 days depending on the type of mabisi production method. The most common fermentation time was 1 day (30%) followed by 2 days (26%) and 3 days (20%). But this variation in fermentation time was also linked to the prevailing ambient temperatures which are influenced by seasons. For instance, during the cold season (June to July) the fermentation took longer as noted by 93% of the respondents by an additional 1–2 days. The endpoint of fermentation was mainly noted by the product becoming thick (55%), tasting sour (10%), formation of whey fraction (13%) and lumpiness (9%) but other observations included the expansion of container (plastic), formation of gas (bubbles), product turning yellowish on top or simply counting the number of days of fermentation. The frequencies of the key processing parameters mentioned above are summarized in Fig 2.

**Fig 3:**
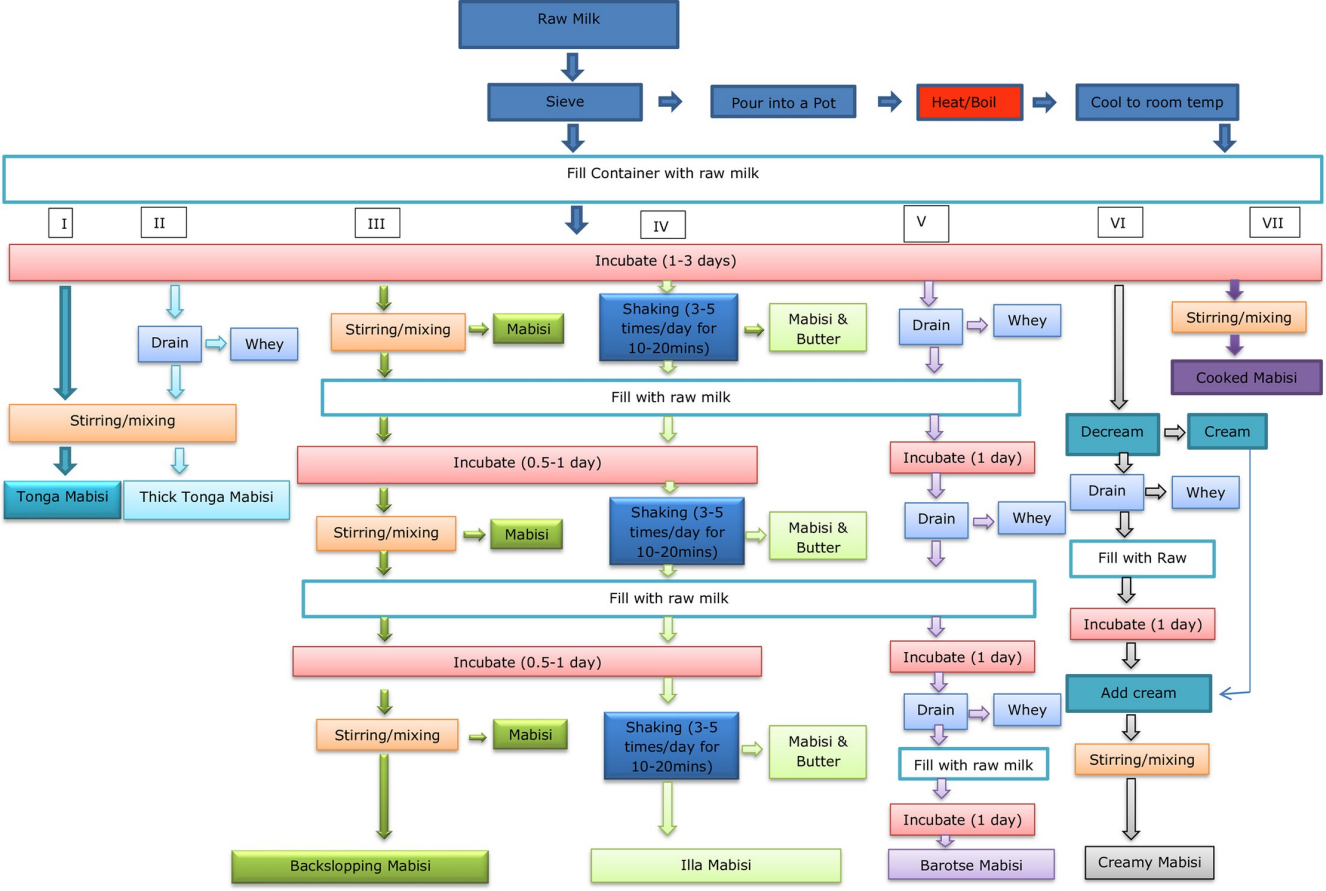
Flow diagram of 7 mabisi production methods. Each method is denoted by a roman numeral from I-VII, which are as follows: I-Tonga, II-Thick tonga, III-Backslopping,IV-Illa, V-Barotse, VI-Creamy and VII- Cooked.

At the end of fermentation, the product is stirred to make it homogeneous and considered ready for consumption thereafter. This process describes the typical production of “tonga type” mabisi which is illustrated in a flow diagram in Fig 3 (process I). However, there is a variation to this method in the case of high whey content in the final product which is usually undesirable. In that case, after fermentation a portion of the whey is drained off before stirring so that a good thickness and homogeneity is retained in the product. The resulting product is referred to as “thick tonga type” of which the production process is illustrated in Fig 3 (process II). It should be stated that until now, the different types of mabisi were all known as “mabisi” or “sour milk” and it became necessary in this study to differentiate the types by coining new names as can be found in this and the following sections. The term tonga type has been derived from the name of the tribe that mostly associated with this method.

The whey fraction has several uses which include: (i) cooking porridge with maize meal, (ii) making “instant mabisi” by adding it to fresh raw milk which coagulates immediately due to its low pH and then giving it to children as a drink and finally (iii) feeding it to dogs and pigs. However, in areas with high milk production during the rainy season from December to April such as Southern and Western provinces, the whey is simply discarded.

#### 3.1.2 Creamy thick type

The production process to make creamy-thick-type of mabisi is illustrated in Fig 3 (process VI). Its production follows the typical tonga-type mabisi production process from raw milk up until the end of fermentation but has a slightly longer fermentation period of usually 2–3 days. Then the cream is removed from the top layer of the product using a spoon and transferred to a separate container such as a cup which is stored in a refrigerator or a cool place. Thereafter, the whey is drained off and the mabisi is allowed to ferment for an additional day after which the cream is added back and mixed with the rest of the product. The final product is creamy and thick, hence the name of the product.

#### 3.1.3 Cooked type

This type of mabisi is produced the same way as the typical tonga type except that there is a cooking step after sieving. The milk is heated in a pot until the foam appears on top and boiling begins, then it is removed from the fire or stove and allowed to cool slowly. Thereafter, it is put in the container for fermentation and left for 2–3 days. Finally, it is stirred and ready for consumption as shown in Fig 3 (process VII).

#### 3.1.4 Backslopping type

The production process for this type of mabisi is shown in Fig 3 (process III). This follows the steps in the typical tonga type production process up to stirring without any removal of whey. A proportion of the product is removed for consumption but a certain amount is left in the fermentation container as starter culture for the next batch. Since milking is done either once or twice a day, the mabisi for consumption is usually all removed at once in the morning from the fermentation vessel leaving some for the next fermentation. Then fresh raw milk is added and allowed to ferment for half to one day. In some cases, by evening the product is ready and can be consumed for dinner and/or breakfast the following morning. The amount removed for consumption depends on the family size and can vary from half of the container to nearly emptying it (approximately 90–95%). At no time during the production, is whey removed and the mixing tends to be quite vigorous making the product less viscous. There is also a special stirrer called *mpeso*, which is basically a stick with three branches at one end and the stirring is done by putting it between the hands then rotating it back and forth so that the branched part breaks the lumps in the product. Traditionally, the cycles of backslopping mabisi production can go on for several months especially when milk production is high during the rainy season because the container is always filled to the top. When milk production is low and the container does not get filled leaving a big gap at the top which may result in an unpleasant smell after approximately 10 days due to proteolytic breakdown of the product on the walls of the container. Once this happens, the container is emptied and subsequently, washed after which the production process can start all over again.

#### 3.1.5 Illa type

This type of mabisi is similar to the backslopping type but it has a churning or agitation process which produces butter granules as illustrated in Fig 3 (process IV). The process starts the same way as the tonga type, once the raw milk becomes sour after a day, the calabash is shaken back and forth either on a mat on the ground or on the laps of the producer. This task was traditionally done by the elderly men, usually a grandfather or the head of the HH. The calabash is shaken for 5–20 minutes each time for several times in a day. If the shaking starts in the morning the product can be ready at noon and should have been shaken at least 3–5 times. This shaking leads to the formation of butter granules which float on top of the mabisi and are removed with a wooden spoon then placed in a small pot or cup. The mabisi is poured out of the calabash but some of it is left as starter culture for the next batch. The raw milk is then added to the calabash which it given a gentle stir and left to ferment for a few hours. Shaking starts again in the late afternoon, into the evening and the following morning. Around mid-morning, the butter and mabisi are removed and raw milk is added again then the process continues. This process is continuous for up to a year or longer. This method of production was given the name ‘Illa type’ because its production was dominated by the Illa speaking people.

The butter is washed with water and stored in a small pot or cup in a cool area of the house. It is mostly used for cooking porridge, vegetables, fish and meat but it can also be used as lotion after a short heating and cooling process. Traditionally, the lotion was used by everyone in the HH but nowadays, it is only used for babies with sensitive skins.

#### 3.1.6 Barotse type

This type of mabisi is mainly produced in the Western province of Zambia (formerly, known as ‘Barotseland’ and hence the name of the product, ‘barotse type’) and illustrated in Fig 3 (process V). It is made in a calabash or plastic container with a small opening on top. The raw milk is placed in a calabash after sieving and left to ferment for 2–3 days until there is clear whey separation. The calabash has a draining hole at the bottom with a plug which is removed to drain the whey and subsequently, plugged back. Once all the whey has been drained, fresh raw milk is added to the calabash without stirring and the lid is closed. The product is left to ferment for a day, after which the draining plug is removed to drain off the newly formed whey. The drain plug is plugged back and fresh raw milk is added again and allowed to ferment for another day. The whey is drained for a third time and fresh raw milk is added again and fermented for another day. Usually at the fourth time of whey draining, there tends to be little or no whey to drain which signifies the end of the process. The resulting product is shaken vigorously and poured out into a bucket or pot. This product is usually very acidic and very thick like a cottage cheese and is called “mafi”. The end point of production differs from producer to producer with some draining the whey only twice whereas others drain several times until there is no more whey being produced which can be up to 4–5 times. This also leads to a slight variation in the thickness of the product. The product is generally called “mabisi yatemile” which means sour milk when it is not as thick as “mafi”. If the product is too thick or too sour, fresh raw milk is added to it and mixed and then it can be consumed. When using a plastic container for fermentation, there is no drain plug at the bottom of the container. So the draining is done by making a small hole through the curd using a thin long stick but usually a fresh grass straw is used and the container is tilted to allow the whey to drain out through the opening in the curd at the top of the container. These containers have a 2.5, 5 and 20 litre capacity. Whey that is removed from the barotse-type mabisi is usually feed to the dogs or simply discarded. During the periods of low milk production, the milk will be added to a container daily and allowed to ferment simultaneously until it is full and that is when the whey is drained and the process continues as described above.

#### 3.1.7 Other practices of coagulating milk

Also other practices were found in Zambia that involve milk coagulation without a fermentation step and strictly speaking cannot be called mabisi. These include adding lemon juice to fresh raw milk which causes the milk to coagulate due to the lowering of the pH of milk to less than 4.6 as a result of the citric acid in the lemon juice. This practice was not only common among herd’s men and boys who coagulated the milk in the bush as a quick meal/snack while looking after the cattle but also in areas where mabisi production was not so popular such as parts of Eastern province. Raw milk is also coagulated by addition of local fruits such as baobab fruit pulp powder to make a product called chivalavati (a product similar to Mutandabota of Zimbabwe [15]), fruit pulp from tamarind (busiika) and nkula(nchenje), the bark of the mukololo tree as well as juice extracted from the roots of a tree called ‘kabodo’. These practices were common in Southern province.

### 3.2 Distribution of the mabisi production methods throughout Zambia

**Fig 4:**
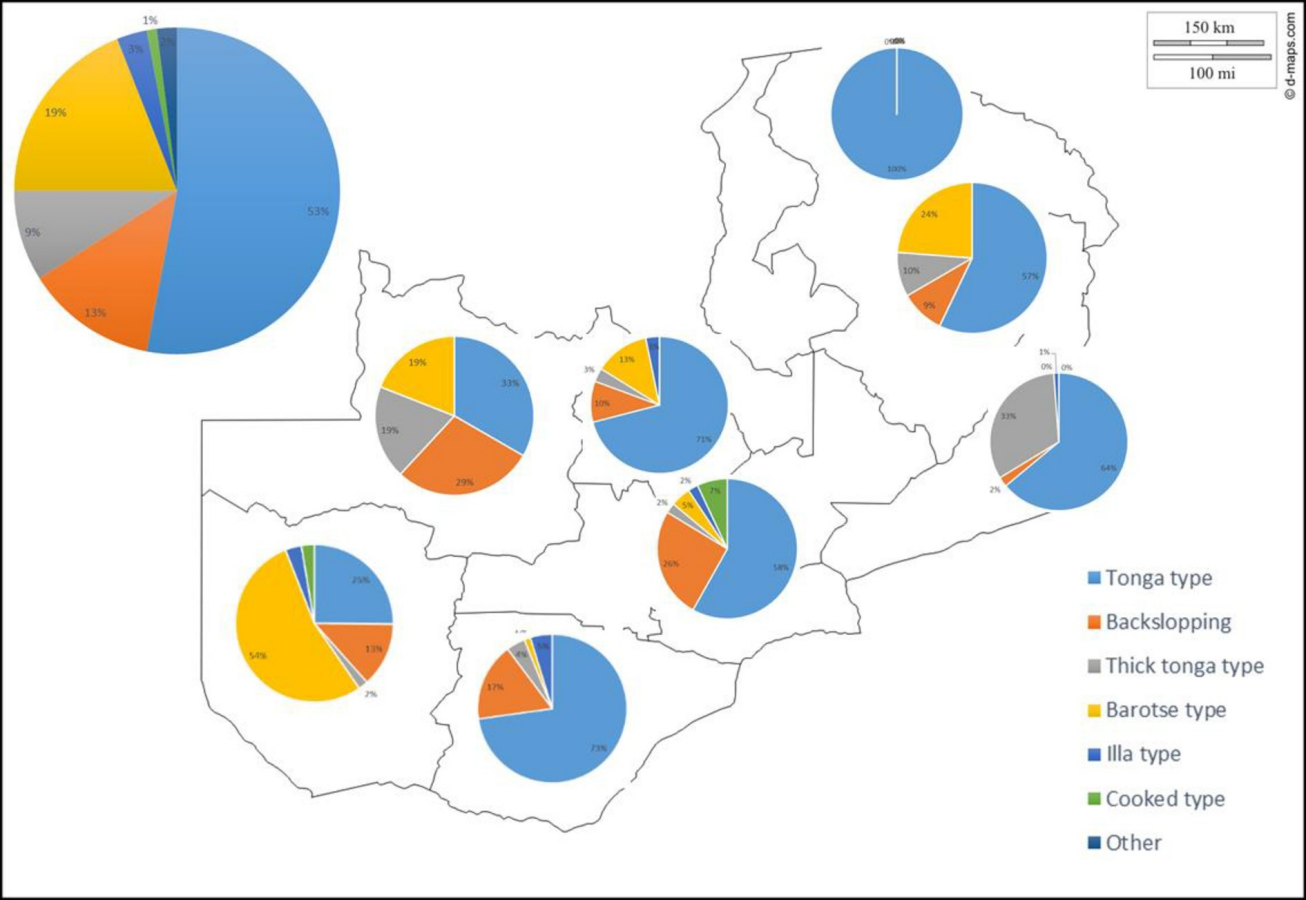
Regional distribution of different mabisi production methods. The large pie chart outside the map shows the overall proportion of the production methods for the country whereas each of the ones inside map shows the regional distribution. (Blank map courtesy of d-maps.com).

The overall proportions of the most frequently used production methods among the respondents are shown in Fig 4 with the tonga type being the most popular followed by barotse type, then backslopping type, with thick-tonga type being fourth and the rest accounting for less than 5% each. The tonga type was produced in all provinces of the country by all the different tribes interviewed but the barotse type was mostly popular in Western province, parts of Southern, Central, Copperbelt, North-western and Muchinga provinces. In Western province, this was practiced in all the districts visited mostly by the Lozi and Nkoya people. Backslopping was widely practiced in Southern, Western and Central provinces but was less popular in Eastern, Muchinga and Copperbelt provinces. The thick-tonga type was found in all provinces studied except for Northern province. The illa type was mainly practiced in Southern and Central provinces mostly among the Illa and Tonga people of Namwala, Choma, Monze and Itezi-tezi districts but it was also practiced to a lesser extent in Western province. The creamy type was exclusively practised on the Copperbelt province (Luanshya district) by different tribes. And lastly, cooked type was practiced in Central (Mkushi district) Western (Kalabo, Kaoma and Sioma districts) and Southern (Monze, Zimba and Sinazongwe districts) provinces by Lala, Lozi/Luvale and Tonga people, respectively.

### 3.3 Distribution of tribes (ethnic groups) that produced mabisi

**Fig 5:**
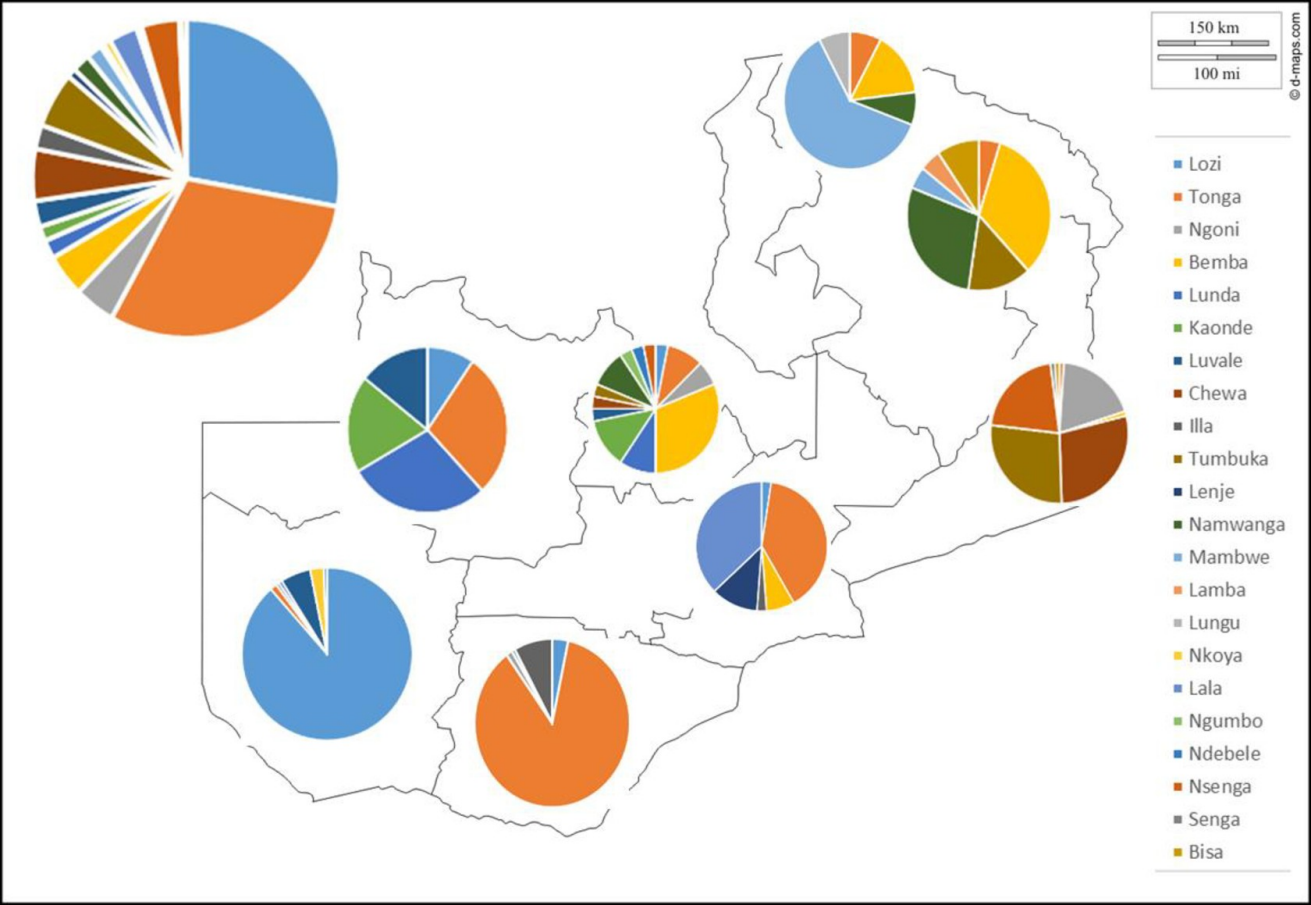
Distribution of proportion of mabisi producing respondents’ tribes (ethnic groups). The overall proportion is shown in the large pie chart outside the map and regional distribution are shown inside in the map. (Blank map courtesy of d-maps.com).

There are a total of 73 tribes in Zambia but the respondents (n = 532) from this survey were only drawn from 22 tribes as depicted in Fig 5. The most predominant tribe that was involved in mabisi production were Tongas followed by Lozis and these are mostly found in the Southern and Western provinces of Zambia, respectively. The Tongas were found in all the provinces but the Lozis were mainly found in Western province. The former were found all over the country due to migration in search of grazing land as well as better agricultural land for crop production in high rainfall regions. The tribes that have a tradition of making mabisi are generally cattle keepers and these include: Tonga, Lozi, Illa, Lenje, Nkoya, Namwanga and Mambwe and mainly originate from Southern, Western, Central and Muchinga provinces. However, despite some tribes from Eastern province also raring cattle, only some Tumbuka and Ngoni people were associated with mabisi production traditionally. Copperbelt province had the most diversity in tribes because it is a mining area which attracts peoples from different regions.

### 3.4 Relationship between production practices and ethnic groups

**Fig 6:**
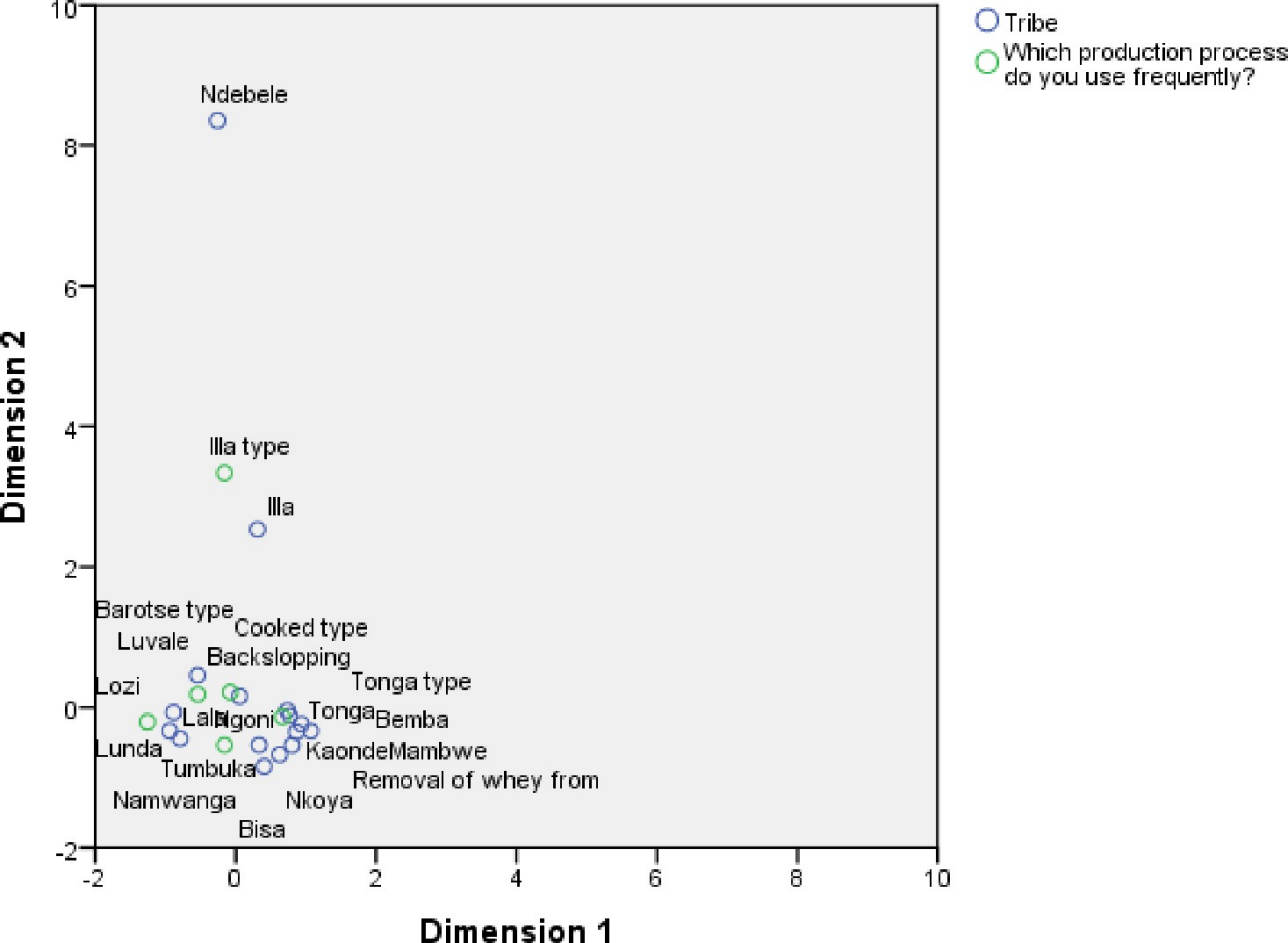
Correspondence analysis (CA) of production practices and tribes (ethnic groups). The fig shows the tribes that practice a certain production method are grouped around it. The production methods in this fig are; tonga, thick tonga (Removal of whey), cooked, illa and barotse types whereas the tribes are listed in Fig 5.

The association between production practices and ethnic groups was analysed using correspondence analysis (CA) which shows that about 9 tribes clustering around tonga type mabisi in Fig 6. Tonga type is different from the barotse type as they are on opposite ends of the first dimension but tonga type with whey removed (thick tonga type) is closer to backslopping, illa and cooked types. The barotse type is strongly associated with Lozi, Luvale and Lunda tribes while the cooked type is associated with the Lala tribe of central province. The tonga type is associated with the Tonga, Bemba, Mambwe, Kaonde, Ngoni and Nkoya tribes. Illa type is associated with the Illa tribe. The second dimension shows that illa type is different from the rest and Ndebele tribe is also more separated from the rest.

### 3.5 Relationship between production practices and geographic location

**Fig 7:**
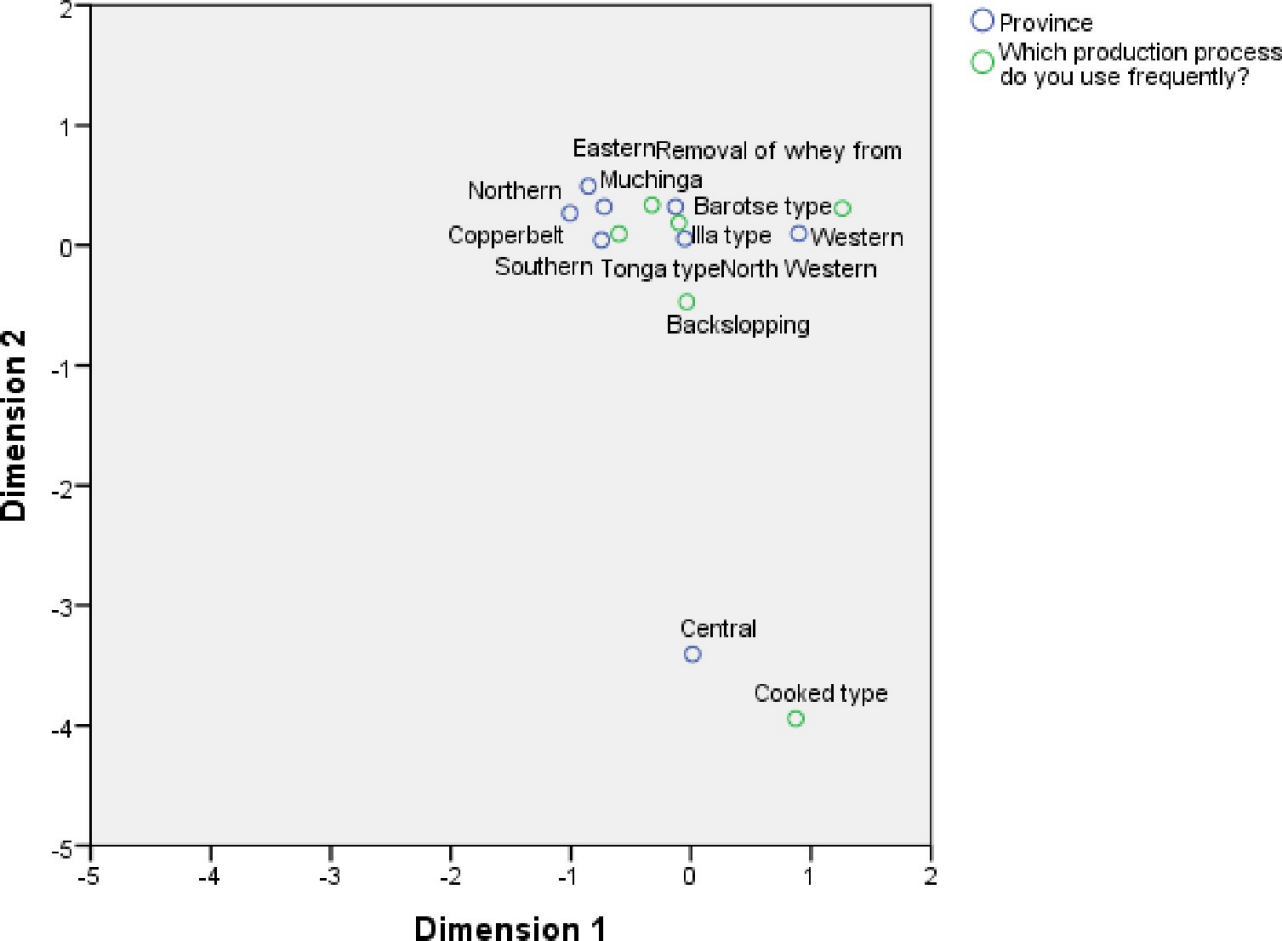
Correspondence analysis of production practices and geographic location. The fig shows provinces grouped around the production methods mostly practiced in their regions.

The association between the production practices and geographic location was also examined using CA and it shows that tonga type and barotse type are different and with the highest degree of differentiation on the first dimension as shown in Fig 7. Tonga type is closely associated with whey removed (thick tonga type), backslopping and illa type as well as several provinces namely: Southern, Copperbelt, Northern, Muchanga and Eastern provinces. Barotse type was associated with Western provinces. On the second dimension, the cooked type was furthest away from the rest and so was Central province from the other provinces but the two were closely associated.

### 3.6 Relationship between production practices and fermentation time

**Fig 8:**
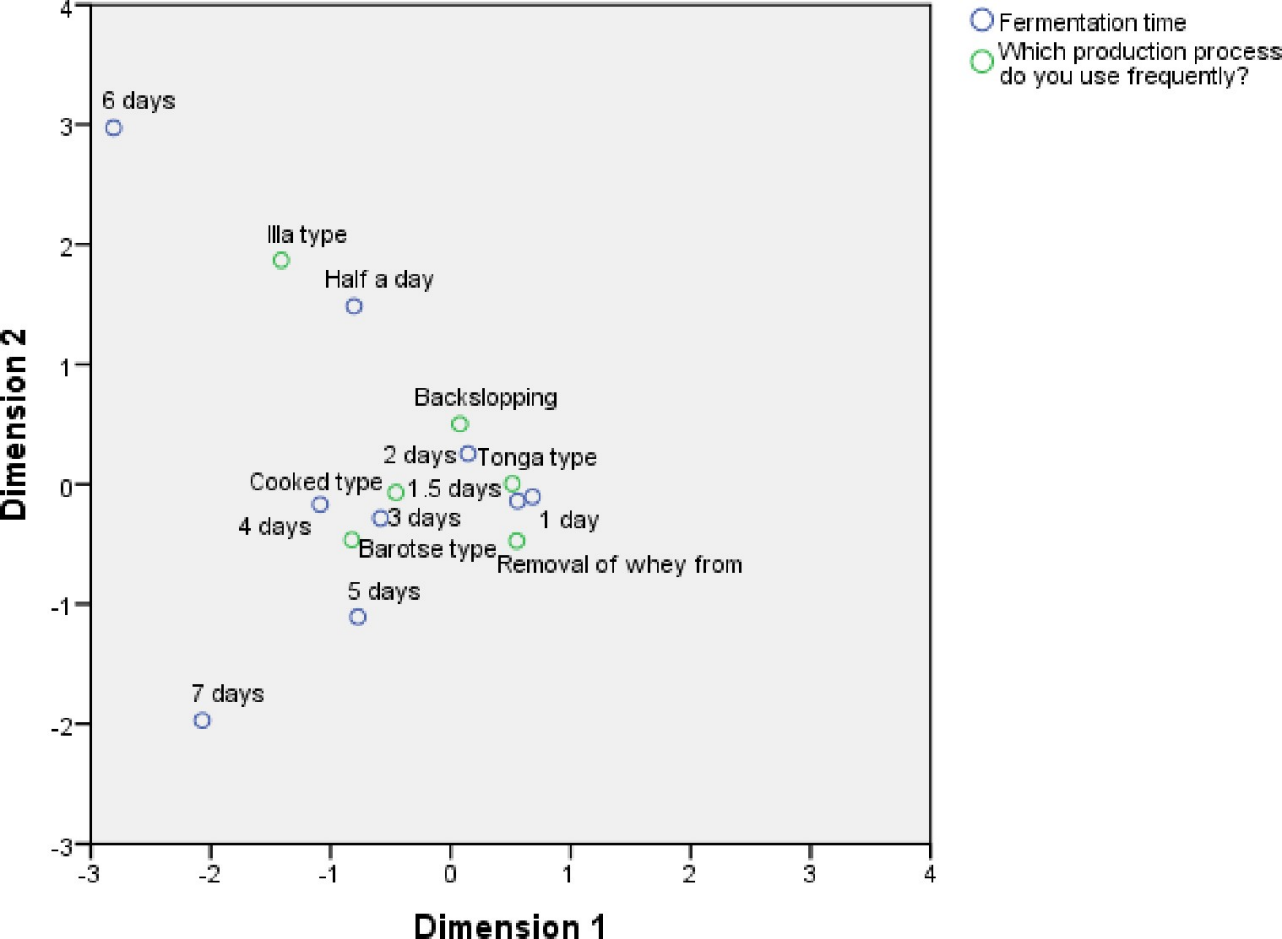
Correspondence analysis of production practices and fermentation time. The fig shows the fermentation time associated with the production methods.

Fermentation time is an important process parameter in mabisi production and its association with production practices was analysed with CA. The results of CA show that barotse and tonga types are furthest from each other on the first dimension with the former closely associated with fermentation times of 4 and 5 days whereas the latter is associated with 1 and 1.5 days (Fig 8). Cooked type is associated with 3 days fermentation time. This can be explained by the fact that this production-type involves a boiling process which kills the initial microflora and therefore the fermentation takes longer incubation times. Dimension 2 shows that illa type is less associated with the rest of the other production practices but is closely associated with the fermentation time of half a day.

### 3.7 Relationship between production practices and type of fermentation container

**Fig 9:**
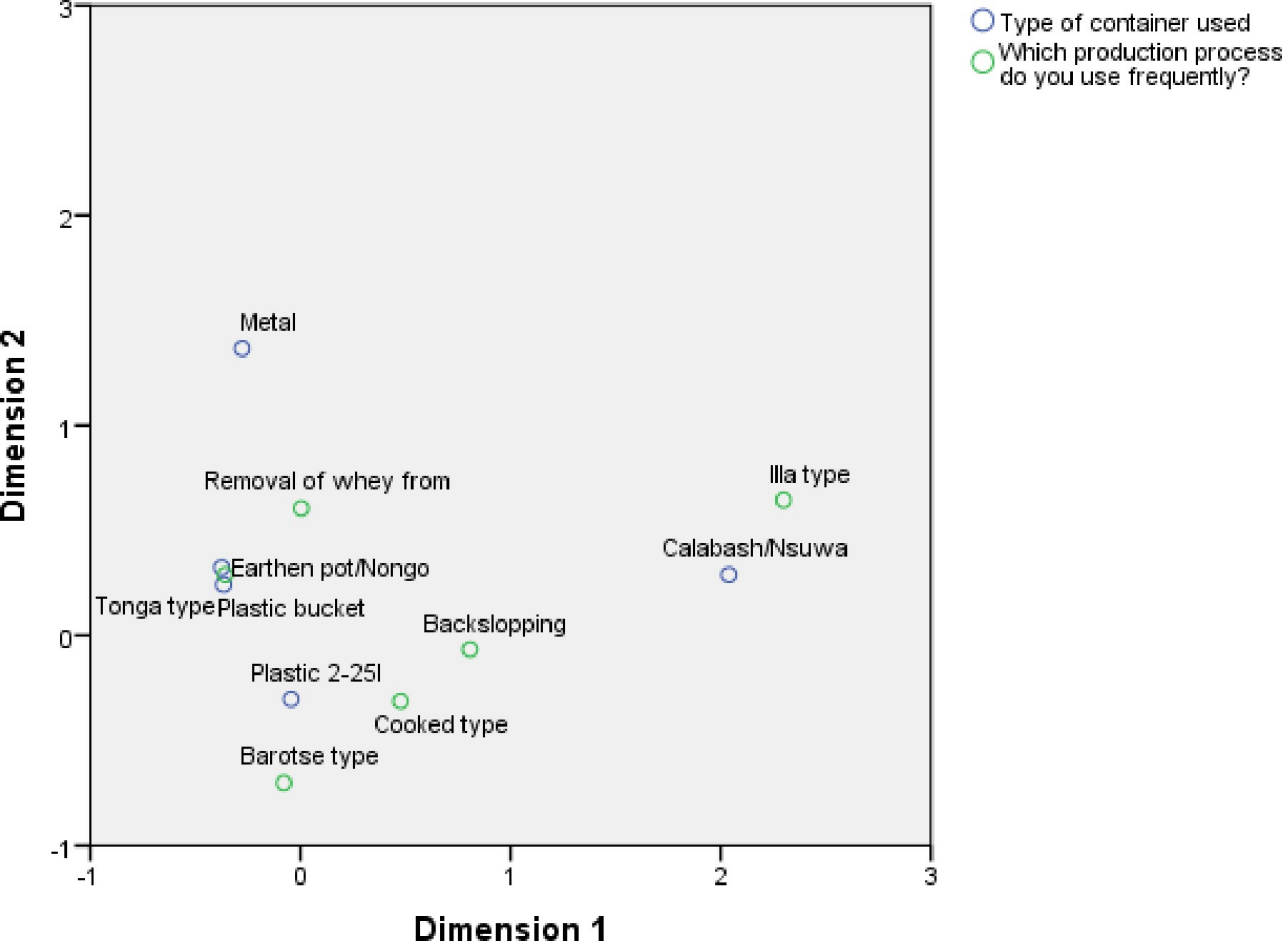
Correspondence analysis of production practices and fermentation container. The fig shows the type of fermentation container linked to a particular mabisi production method.

The type of fermentation container (shown in Fig 1) is another important process parameter and the association with production practices was also analysed with CA as shown in Fig 9. We found that tonga type mabisi is associated with plastic bucket and earthen pot while the plastic container with a small opening at the top is associated with the barotse, cooked and backslopping types. The illa type is closely associated with the calabash. The metal container is furthest apart from the rest but only closest to the whey removal (thick tonga type) as shown on dimension 2.

### 3.8 Hygiene practices

**Fig 10:**
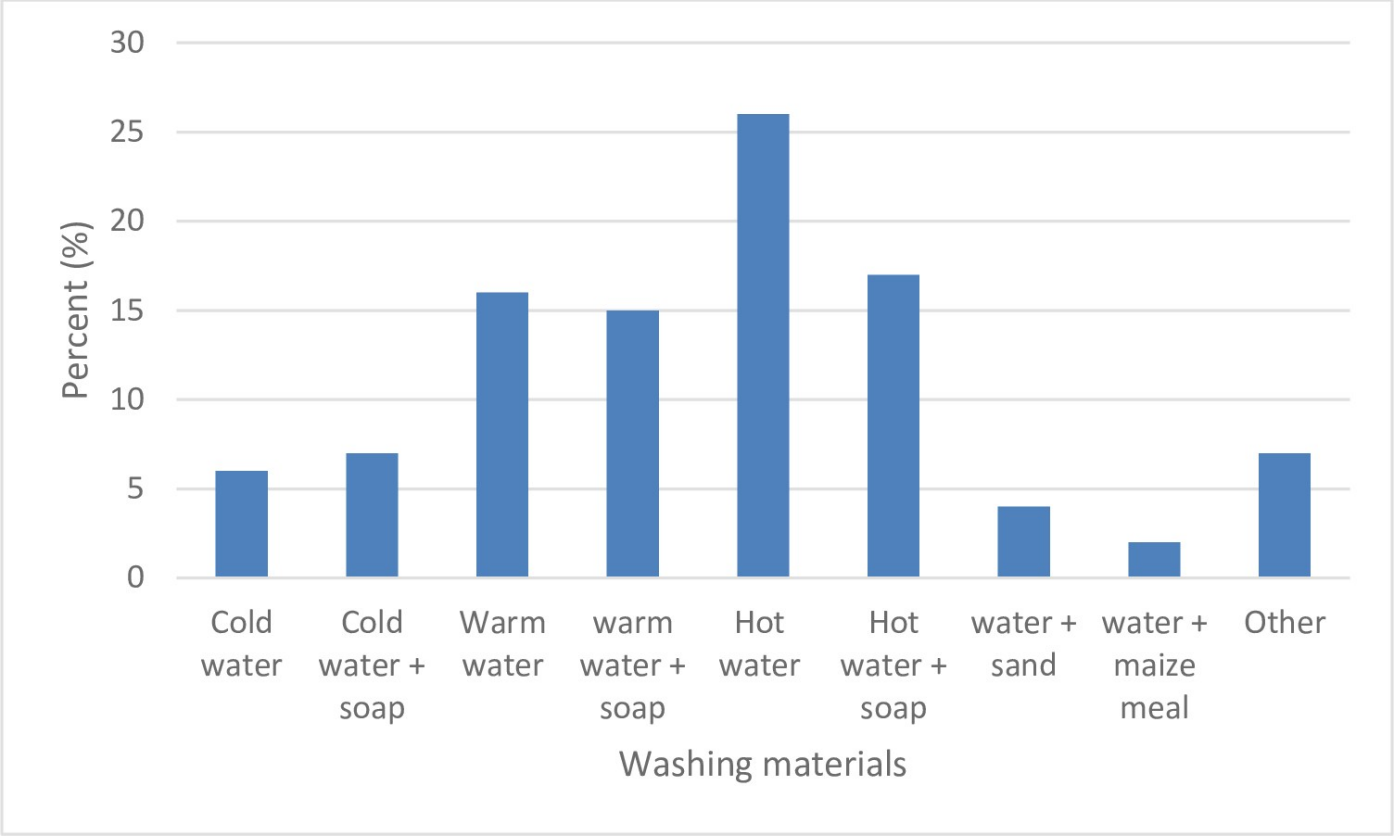
Washing practices of fermentation containers and their proportionate use.

Mabisi is made using different types of containers which include: calabash, plastic buckets, metal cans/pots and earthen pots. Since these containers are made from different types of material, the cleaning method will have different effects on each container. Ninety four percent (94%) of the respondents reported that they washed their containers at the end of mabisi production depending on the type of production method used. Out of these, the highest proportion of up to 48% washed their containers with water only, out of which 26% used hot water, 16% used warm water and 6% used cold water as shown in Fig 10. The total proportion of the producers that used detergent to wash their container was 39% with 17% of them using hot water, 15% using warm water and 7% using cold water. From the remainder, 3% used water with sand, 1% used water with ash, 2% used water with maize meal to wash their containers. These different ways of cleaning the fermentation container are likely to have different effects on the surface of the container which might lead to the formation of biofilms in some cases, [16]. With the fermentation mostly done in the same container and with over half of the producers only washing their containers with water, there is a high likelihood of biofilm formation in most of these containers which eventually play a part in subsequent fermentations as well as quality and safety of the final product. The main reason for some of the respondents not to use detergent to wash their containers was because of residual odours of the detergent in the product which was regarded as extremely undesirable.

### 3.9 Uses of mabisi

Mabisi is mostly consumed directly by drinking or mixed with a thick or light maize porridge. The thick maize porridge locally called ‘nshima’ is a staple food and can also be made from other cereal crops as well such as sorghum and millet or in some cases a blend of maize and cassava meal. This thick porridge is eaten with mabisi for breakfast and lunch or as a dessert after lunch or dinner. The light porridge with mabisi is mostly eaten for breakfast especially by the children. Mabisi can also be eaten with boiled grains of sorghum, millet, whole grain maize (magwaza), dehulled maize (samp) or grits (masembe for cooking porridge). Other cereals include rice and pasta but the latter is only consumed in a few urban areas. Root and tuber crops such as cassava, sweet potato and irish potato can be boiled and eaten with mabisi as a main meal. Mabisi is also consumed with both local and exotic fruits as well as different types of pumpkins. Furthermore, it is also consumed with baked products, cooked legumes and used during cooking of dishes such as meat, fish and vegetables. The various types foods that are consumed with mabisi are shown in Table 3.

**Table 3:** Foods consumed with mabisi.

Product category	Type
Cereals	Maize porridge from meal or grits (chelee, Illya)* Sorghum/millet porridge (chelee)* Boiled immature sorghum grains called ‘mututu’ (muselu)* Boiled whole maize grain (titili, dwobo, chibobole, magwaza)* Dehulled maize (samp, musohya)* Boiled fresh maize cob Roasted maize grains called ‘chiwaya’ Rice Pasta Fermented cereal drink called ‘chibwantu’ Opaque beer (sibamu)*
Fruits	Pawpaw, boiled unripe mango, avocado, baobab powder (chivalavati, mulondo)*, tamarind (mpalwe)*, masuku (chimvwe, chilimbu)*, banana, ripe mango
Pumpkins	Yellow & green pumpkins Green pumpkin with boiled fresh maize (chidyobo)*
Baked confectionery	Bread & buns (chikandi)* Scones
Tubers	Boiled cassava, sweet potatoes, & irish potatoes
Legumes	Beans Beans with rice Roasted groundnuts called ‘musuka’
Cooked side dishes	Beef Fish (tilapia, tigerfish) Vegetables

*Noted the local name of the product eaten with mabisi is indicated in brackets

## 4. Discussion and conclusion

This study shows that there are at least seven different methods of producing mabisi in Zambia. This complements the study by Schoustra [3], who only reported on one prevailing production method, which we have now coined as tonga type mabisi. This tonga type is the most popular amongst all ethnic groups and found throughout the country which suggests that it is acceptable for a broad range of consumers. The production process of the thick tonga type shows that it only differs from the tonga type by the step of whey removal which in turn produces thicker product and suggests that the two products are quite similar in characteristics. The creamy type has a skimming step in addition to whey removal and generally produces a thick creamy product. The latter production method has some similarities with amasi of Zimbabwe which is also produced by a method that involves whey removal and addition of fresh cream to the product producing a thick product, [17].

The cooked type involves a heating and cooling step before fermentation and was the least practiced. However, it was widely practiced in Mkushi district of Central province among the Lalas who reported that they learnt the technique of mabisi making from the Tongas that migrated there from the early 1990s though the latter did not heat their raw milk but made the tonga and backslopping types instead. This practice can be attributed to the public health messages from the Ministry of Health that advocates boiling raw milk before consumption as a way of preventing foodborne infections from raw milk consumption. This practice was also observed in Monze and Sinazongwe districts but to a much lesser extent. A similar product called “mursik” from Kenya has been reported by Nduko [10] although, the other one called “amabere amaruranu” involves both boiling and backslopping. The Illa type and backslopping type are similar as they both involve backslopping steps and several fermentation cycles though the former also has an additional agitation step which leads to the production of butter. This method produces a buttermilk-like product which might be similar to aewsso from Ethiopia [13], kivuguto of Rwanda [11] and nunu of Ghana, [12]. The barotse type involves the repeated alternate removal of whey and the addition of raw milk which eventually produces a very thick and sour product which is sometimes referred to as “mafi”. This name is also used to refer to a type of traditional fermented milk product from South Africa [7, 18]. Further, the practice of continuously adding daily batches of raw milk to a container until it is full and removing the whey thereafter is reported for a product which is also called “mabisi” from Northern Namibia, [8, 19]. This region is close to the Western province of Zambia where the barotse type is most popular. One explanation could be migration of tribes during the Mfecani wars in the time of King Shaka of the Zulu people of present day South Africa in the 1820s [20]. Some displaced tribes settled in parts of Zimbabwe, Botswana, Namibia, Zambia, Malawi and Mozambique. In Zambia for example, the Makololo tribe crossed the Zambezi river and settled in present day Western province [21] while the Ngoni went east and settled in Eastern province [20] but this production practice is not common there. A product called “omashikwa” from Namibia is reported [8, 9] to be made by fermenting raw milk with a root which can be similar to the other methods of coagulating milk reported in this study where roots and tree barks were used.

This study also suggests that migration of ethnic tribes can spread production practices (technology transfer) and consumption of certain foods to other regions. For example, the Tongas migrated to Central (Mkushi) and Muchinga (Mpika) provinces and most likely introduced the tonga type mabisi. However, some production practices can also evolve as a result of this as was observed with cooked type. There can also be barriers to adopting other production practices such as Illa and backslopping that were also practiced by the tongas which may be related to strong consumer preferences. This is an area that requires further investigation. Furthermore, our study also suggests that barotse type mabisi only spread to parts of Southern and Central province that are proximal to Western province which may suggest that the Lozis migrated less or adapted quickly to the tonga type when they did. The study also points out that the Lalas and Bembas (from the Northern parts) were more receptive to mabisi than the Chewas, Nsenga, Senga (from Eastern Province). However, a comprehensive study needs to be undertaken to ascertain these dynamics.

The most important mabisi production parameters observed were the type of containers, fermentation time and temperature, backslopping, agitation, removal of whey, addition of raw milk, heating and cooling, and washing method. All these have a bearing on the quality of the final product which need further investigation. The use of calabashes and earthen pots for fermentation of milk have been reported in other studies [7, 10, 14, 22] but an increased use of plastic and metal containers has been observed in this study largely due to their robustness and availability though their effects on the quality of final product are not known. Fermentation times ranging between 1 and 2 days have been reported in some traditional fermented dairy products [3, 17]. This study shows that fermentation time is a function of temperature as clearly shown with increasing fermentation times by 1 to 2 days during the cold season. The effect of fermentation time and temperature on the quality of mabisi is largely unknown.

Mabisi is produced through a spontaneous fermentation process which involves microbes from the raw milk, containers and immediate environment. These microbes repeatedly produce mabisi in different locations using a variety of production methods whose microbial profiles are essentially unknown. In case of backslopping, there is a transfer of the microorganisms involved from one batch to another and this has been reported in different fermented products, [23, 24]. However, the optimal transfer ratio for backslopping in mabisi production and its effect on product quality is not known. The removal of whey reduces the liquid fraction in the product and leads to the production of highly viscous product such as the thick tonga and barotse mabisi types. The whey contains lactose which is the energy source for the microbes and may influence the microbial composition and quality of the product. This however requires a comprehensive investigation to determine the type of bacteria present which can later be used to develop starter cultures for industrial mabisi production. Food safety is of paramount importance especially for a product that is produced by spontaneous fermentation of raw milk which in some cases is produced under poor hygiene conditions. Therefore, it is imperative that the safety of mabisi needs to be assessed.

In conclusion, seven different methods of producing mabisi were found throughout Zambia. The main process parameters were found to be fermentation time and temperature, type of containers, presence or absence of backslopping, agitation, heating and cooling, removal of whey and addition of raw milk, and washing method. Ethnicity, culture and location have a bearing on the type of production method used, highlighting the effect of regional migration and adopting new foods and food production methods. Mabisi is quite versatile in terms of usage as it can be consumed with a wide variety of foods. However, further studies need to be conducted on microbial composition of the different types of mabisi, production process optimisation, starter culture development, product quality and safety, and consumer perception and preferences. This study shows diverse traditional technologies for fermented milk, which are similar with certain products from other parts of Africa and this underpins the importance of carrying out systematic research in product development and up-scaling production of these products. These products have the potential to reduce food and nutrition insecurity and improve livelihoods of local communities. With more research, mabisi has the potential grow in statute on the African continent and beyond to the level of cheese in the world.

## Supporting information

S1 Questionnaire Questionnaire for Key Informants on the Different Methods of Processing Mabisi. (DOCX)

S1 Checklist Focus Group Discussion (FGD) Checklist of Questions. (DOCX)

S1 Dataset Raw Dataset. (SAV)
