# Supplementary material for: The art of mabisi production: A traditional fermented milk
Source: PLoS One. 2019 Mar 14;14(3):e0213541. doi: 10.1371/journal.pone.0213541 (PMC6417723; doi:10.1371/journal.pone.0213541)
Supplement: S1 Questionnaire — (DOCX) [file pone.0213541.s001.docx]

**S1. Questionnaire. Questionnaire.**


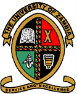

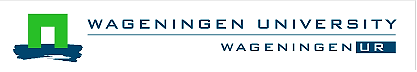


**Questionnaire for Key Informants on the Different Methods of Processing Mabisi**

Dear Respondent,

I am ………………………………………………………………………, an enumerator for the mabisi processing survey. This survey aims at collecting indigenous knowledge on different ways of producing mabisi and the factors that influence its quality with a view of improving and optimizing its production at different scales for local communities. This study is part of a sandwich PhD research project under the University of Zambia in collaboration with Wageningen University of The Netherlands being carried out by Himoonga Bernard Moonga. We thank you for your participation.

**Questionnaire No. ………….**

1. **Questionnaire administration information**

| **N°** | **Questions** | **Answers** | **Code** |
| --- | --- | --- | --- |
| 1.1 | Name of enumerator |  |  |
| 1.2 | Name of respondent |  |  |
| 1.3 | Date of interview |  |  |
| 1.4 | Time | Start :  Finish : |  |
| 1.5 | Location | Village....................................................................  Camp.....................................................................  Block...................................................................  District.................................................................  Province............................................................... |  |
| 1.6 | Contact Number (Mobile phone) |  |  |
| 1.7 | Main language used by respondent | 1. Lozi 2. Tonga 3. Nyanja 4. Bemba 5. Lunda 6. Kaonde 7. Luvale 8. Other (specify)............................................... |  |
| 1.8 | Was a translater used ? | 1. Yes 2. No |  |
| 1.9 | What activities are you involved in with respect to mabisi? | 1. Trader 2. Processor 3. Consumer |  |
|  |  |  |  |

1. **Socio- demographic information**

| **N°** | **Questions** | **Answers** | **Code** |
| --- | --- | --- | --- |
| 2.1 | Gender | 1. Male  2. Female |  |
| 2.2 | Location | Village....................................................................  Camp.....................................................................  Block...................................................................  District.................................................................  Town....................................................................  Province............................................................... |  |
| 2.3 | Ethnic group (Tribe) | 1. Lozi 2. Tonga 3. Nyanja 4. Bemba 5. Lunda 6. Kaonde 7. Luvale 8. Other (specify)................................................... |  |
| 2.4 | Marital status | 1. Single  2. Married  3. Divorced  4. Widow/ widower |  |
| 2.5 | Age in years | 1. ≤18  2. 19-29  3. 30-39  4. 40-49  5. 50-59  6. 60+ |  |
| 2.6 | Education level | 1. No education  2. Primary (Grade 7)  3. Secondary (O-Level or A-Level)  4. Vocational training  5. Tertiary (diploma or degree)  6. Other (specify)......................................................................... |  |
| 2.7 | Who is head of the household? | 1. Father 2. Mother 3. Child 4. Grandmother 5. Grandfather 6. Aunt 7. Uncle 8. Other (specify)....................................................................... |  |
| 2.8 | Size of the Household | 1. 0-6 2. 6-10 3. >10 4. Other (specify) …………………………………………………………………. |  |
| 2.9 | What kind of livestock do you rear? | 1. Cattle (#.…….) 2. Goats (#..……) 3. Sheep (#………) 4. Poultry (#…….) 5. None |  |

1. **Mabisi processing methods**

| **N°** | **Questions** | **Answers** | **Code** |
| --- | --- | --- | --- |
| 3.1 | Where do get your raw milk? | 1. Own cows  2. Buy from neighbours  3. Buy from markets  4. Other (specify)………………………………………………………………….. |  |
| 3.2 | Do you sieve the milk? | Yes  No |  |
| 3.3 | If Yes, what material do you use for sieving? | 1. Cloth 2. Plastic strainer 3. Metal strainer 4. Other (specify)......................................................................... |  |
| 3.4 | What containers do you use for mabisi making? | 1. Plastic bucket 2. Plastic (2l) 3. Metal pot 4. Calabash (Nsuwa) 5. Earthen pot (Nongo) 6. Other (specify)…………………………………………………………………. |  |
| 3.5 | Where do you ferment/incubate your milk? | 1. In the house 2. In the outside kitchen 3. Outside in the sun 4. Outside in the shed 5. Other (specify) ………………………………………………………………. |  |
| 3.6 | How long do you ferment the milk? | 1. Half a day (12hrs) 2. One day 3. One & a half day 4. Two days 5. Three days 6. Other (specify).................................................................... |  |
| 3.7 | Is the fermentation period longer in the cold season? | 1. Yes 2. No |  |
| 3.8 | If Yes, how much longer? | 1. Half a day more 2. One more day 3. Two more days 4. Three more days 5. Other (specify) ………………………………………………………………… |  |
| 3.9 | How do you tell that mabisi is ready for consumption? | 1. It becomes thick 2. Tastes sour 3. Form whey fraction (water separates) 4. Forms lumps 5. Other (specify)………………………………………………………………… |  |
| 3.10 | Do you remove whey (water) from mabisi? | 1. Yes 2. No 3. Sometimes 4. Other (specify)………………………………………………………………… |  |
| 3.11 | If Yes, do you add fresh raw milk to your mabisi afterwards? | 1. Yes 2. No |  |
| 3.12 | If Yes, how much longer do you ferment your mabisi? | 1. Half a day 2. One day 3. One & a half days 4. Two days 5. Other (specify) …………………………………………………………………. |  |
| 3.13 | Do you remove whey again before consumption? | 1. Yes 2. No |  |
| 3.14 | If Yes, how many times do repeat this process before consumption? | 1. Once 2. Twice 3. Thrice 4. Other (specify)………………………………………………………………….. |  |
| 3.15 | Do you shake the mabisi container during fermentation? | 1. Yes 2. No 3. Once at the end of fermentation 4. Other (specify) |  |
| 3.16 | If Yes, do you find butter granules (fat lumps) in the mabisi? | 1. Yes 2. No |  |
| 3.17 | If Yes, what do use them for? | 1. Cooking relish 2. Cooking porridge 3. Eating with bread 4. Use as body lotion 5. Other (specify)………………………………………………………………… |  |
| 3.18 | Do you wash your container after making mabisi? | 1. Yes 2. No |  |
| 3.19 | If Yes, what do you use to wash it? | 1. Cold water only 2. Cold water with soap 3. Warm water only 4. Warm water with soap 5. Hot water only 6. Hot water with soap 7. Other (specify)………………………………………………………………. |  |
| 3.20 | Do you use backslopping (put old mabisi in fresh milk to start the fermentation)? | 1. Yes 2. No |  |
| 3.21 | If Yes, how long is the fermentation? | 1. Half a day 2. One day 3. One & a half days 4. Two days 5. Three days 6. Other (specify) ……………………………………………………………….. |  |
| 3.22 | Which container has longest fermentation time? | 1. Plastic bucket 2. Plastic (2 litre) 3. Metal pot 4. Calabash (Nsuwa) 5. Earthen pot (Nongo) 6. Other (specify) ……………………………………………………………….. |  |
| 3.23 | Which mabisi making process do you use frequently? | 1. Fresh raw milk fermented in a regular container each time 2. Backslopping (mixing mabisi with fresh raw milk every time) 3. Removing whey after fermentation and stirring 4. Removing whey, adding fresh raw milk & fermenting more 5. Shaking the container during fermentation and removing butter granules (fat lumps) 6. Other (specify) …………………………………………………………………. |  |
| 3.24 | What volumes of mabisi do you produce? | 1. <2 litres 2. 2-5 litres 3. 5-10 litres 4. 10-20 litres 5. >20 litres 6. Other (specify)………………………………………………………………… |  |
| 3.25 | Do you sell mabisi? | 1. Yes 2. No |  |
| 3.26 | Who buys your mabisi? | 1. Traders 2. Neighbours 3. Other (specify) ……………………………………………………………….. |  |
