# Supplementary material for: The art of mabisi production: A traditional fermented milk
Source: PLoS One. 2019 Mar 14;14(3):e0213541. doi: 10.1371/journal.pone.0213541 (PMC6417723; doi:10.1371/journal.pone.0213541)
Supplement: S1 Checklist — (DOCX) [file pone.0213541.s002.docx]

**S1. Checklist. Focus Group Discussion(FGD) Checklist of Questions**

**A. Socio-demographic information**

1. Name
2. Sex
3. Age
4. Ethnicity (tribe)
5. Marital status
6. Education level
7. Household (size, position in the household)
8. Source of livelihood
9. Location – province, district, block, camp, village
10. Number of cattle, goats & sheep owned
11. Language of interview
12. Contact details – mobile phone number & email

**B. Processing, product quality & storage**

1. Do you produce Mabisi?
2. How long have you been producing?
3. Who taught you how to produce mabisi?
4. Is your current mabisi making process the same as your parents?
5. Do you produce mabisi throughout the year?
6. How much do you produce per day/week/month? (in litres)
7. Where do you produce from, at home? Elsewhere?
8. Where do you get the raw milk? Seasonal variation?
9. What type of raw milk do you use? Cow or goat’s milk?
10. What are the steps in the processing?
11. What equipment is used? Types of containers?
12. Do you inoculate your raw milk?
13. Where do you incubate the product? (place)
14. How long does it take to produce mabisi?
15. Is the incubation period the same throughout the year?
16. Does the container affect incubation period?
17. How do you know that mabisi is ready?
18. Do you drain the whey?
19. Do you stir the mabisi when it is ready?
20. Do you shake it during production?
21. What are the critical steps/important steps during production? Bottlenecks where things may go wrong?
22. Is the product quality constant each time you produce?
23. What is good Mabisi? What do they do to control quality? Any grading #1? #2?
24. How many types of mabisi can you make?
25. How long can you keep the Mabisi (also considering distant markets)?
26. Where/how do you store your mabisi?
27. Does the processing fail sometimes?
28. How do you correct or avoid this from happening?
29. How did they start the first batch?
30. Do you exchange with other producers?
31. Are other producers competitors or colleagues?
32. Whose involved in the production of mabisi? Men or Women or Children?
33. Are there shared roles during the process?
34. How does the type of breeds of cows affect the quality of mabisi?
35. Does the type of management of cows (intensive/free) affect the quality of mabisi?

**C. Consumers and Sales**

1. Where/how do you sell?
2. Who buys the product?
3. What type of Packaging do you use?
4. What is the price per cup or 2 litre container?
5. How much do you sell per day?
6. What profit do you make? Big or small?
7. What do the consumers like about your Mabisi?
8. Do they prefer your mabisi or that of other producers?
9. What do the consumers use (eat/drink) mabisi for?
10. What time of the day is it consumed?
11. Do consumers come back and buy again?
12. What the frequency of buying or how good is consumer retention?
13. What quality criteria do the consumers look for?
14. Does your product face competition from commercial products?
15. What ambitions do you have for commercializing your production?
16. How can you increase your sales or access to other markets?
